# Changes in stiffness of the extracellular and pericellular matrix in the anulus fibrosus of lumbar intervertebral discs over the course of degeneration

**DOI:** 10.3389/fbioe.2022.1006615

**Published:** 2022-12-23

**Authors:** Sebastian Höflsauer, Florian Christof Bonnaire, Charlotte Emma Bamberger, Marina Danalache, Martina Feierabend, Ulf Krister Hofmann

**Affiliations:** ^1^ Laboratory of Cell Biology, Department of Orthopaedic Surgery, University Hospital of Tübingen, Tübingen, Germany; ^2^ Medical Faculty of the University of Tübingen, Tübingen, Germany; ^3^ Department of Orthopaedic Surgery, University Hospital of Tübingen, Tübingen, Germany; ^4^ Institute for Bioinformatics and Medical Informatics, Faculty of Science of the University of Tübingen, Tübingen, Germany; ^5^ Department of Orthopaedic Trauma and Reconstructive Surgery, RWTH Aachen University Hospital, Aachen, Germany

**Keywords:** intervertebral disc, degeneration, spatial chondrocyte organisation, extracellular matrix, pericellular matrix, atomic force microscopy

## Abstract

Analogous to articular cartilage, changes in spatial chondrocyte organisation have been proposed to be a strong indicator for local tissue degeneration in the intervertebral disc (IVD). While a progressive structural and functional degradation of the extracellular (ECM) and pericellular (PCM) matrix occurs in osteoarthritic cartilage, these processes have not yet been biomechanically elucidated in the IVD. We aimed to evaluate the local stiffness of the ECM and PCM in the anulus fibrosus of the IVD on the basis of local chondrocyte spatial organisation. Using atomic force microscopy, we measured the Young’s modulus of the local ECM and PCM in human and bovine disc samples using the spatial chondrocyte patterns as an image-based biomarker. By measuring tissue from 31 patients and six bovine samples, we found a significant difference in the elastic moduli (E) of the PCM in clusters when compared to the healthy patterns single cells (*p* = 0.029), pairs (*p* = 0.016), and string-formations (*p* = 0.010). The ECM/PCM ratio ranged from 0.62–0.89. Interestingly, in the bovine IVD, the ECM/PCM ratio of the E significantly varied (*p* = 0.002) depending on the tissue origin. Overall the reduced E in clusters demonstrates that cluster formation is not only a morphological phenomenon describing disc degeneration, but it marks a compromised biomechanical functioning. Immunohistochemical analyses indicate that collagen type III degradation might be involved. This study is the first to describe and quantify the differences in the E of the ECM in relation to the PCM in the anulus fibrosus of the IVD by means of atomic force microscopy on the basis of spatial chondrocyte organisation.

## Introduction

Degeneration of the intervertebral disc (IVD) is a leading cause for low back pain (Zheng and Chen, 2015). The Global Burden of Disease study 2015 identified low back pain as the main factor for disability worldwide (Hartvigsen et al., 2018). Conservative treatment remains symptomatic with pain management and physiotherapy, and in severe cases fusion surgery of the affected motion segment is performed. The original architecture and function of the disc can, however, not be restored.

The IVD consists anatomically of three major components (Humzah and Soames, 1988): 1) The anulus fibrosus forms the outer part of the disc and consists of 15–25 circular lamellae (Marchand and Ahmed, 1990) composed of mainly collagen type I (Eyre and Muir, 1977), II, III, V, VI, IX, and XI (Roberts et al., 1991). 2) In the centre of the disc lies the nucleus pulposus. Its fibre network mostly consists of collagen type II (up to 85%) (Eyre and Muir, 1977) and it is rich in proteoglycans that are responsible for generating a high osmotic pressure attracting water. 3) The cartilaginous end plates seclude the IVD in caudal and cranial direction. This complex architecture encompassing all the different components is needed for the dual function of the IVD as a motion segment and a shock absorber.

Microscopically, the chondrocytes forming only 1% of the disc’s volume (Bibby et al., 2001), are scarcely dispersed within a vast and dense extracellular matrix (ECM) (Roberts et al., 1991). Directly encompassing the individual cells, a distinct substructure of the ECM is present. It is termed the pericellular matrix (PCM) and it is rich in collagen type III and VI (Wu et al., 1987; Roberts et al., 1991) and proteoglycans (Roberts et al., 2006) such as perlecan (Melrose et al., 2008). Even though the function of the PCM is still not yet completely elucidated, preliminary data suggest that it plays a role in the mechanoprotection of the chondrocytes from overload (Peters et al., 2011; Hofmann et al., 2018) and as a mechanical signal transducer by cell-matrix interactions (Guilak et al., 2006).

During IVD degeneration, catabolic processes and changes in cell synthesis lead to a reduction in water-binding proteoglycans (Dowdell et al., 2017). Losing its original matrix architecture, the nucleus shrinks becoming more fibrotic. Such morphological changes are in turn responsible for increasing stress to which the anulus is subjected during loading thus further propagating degeneration.

**FIGURE 1:**
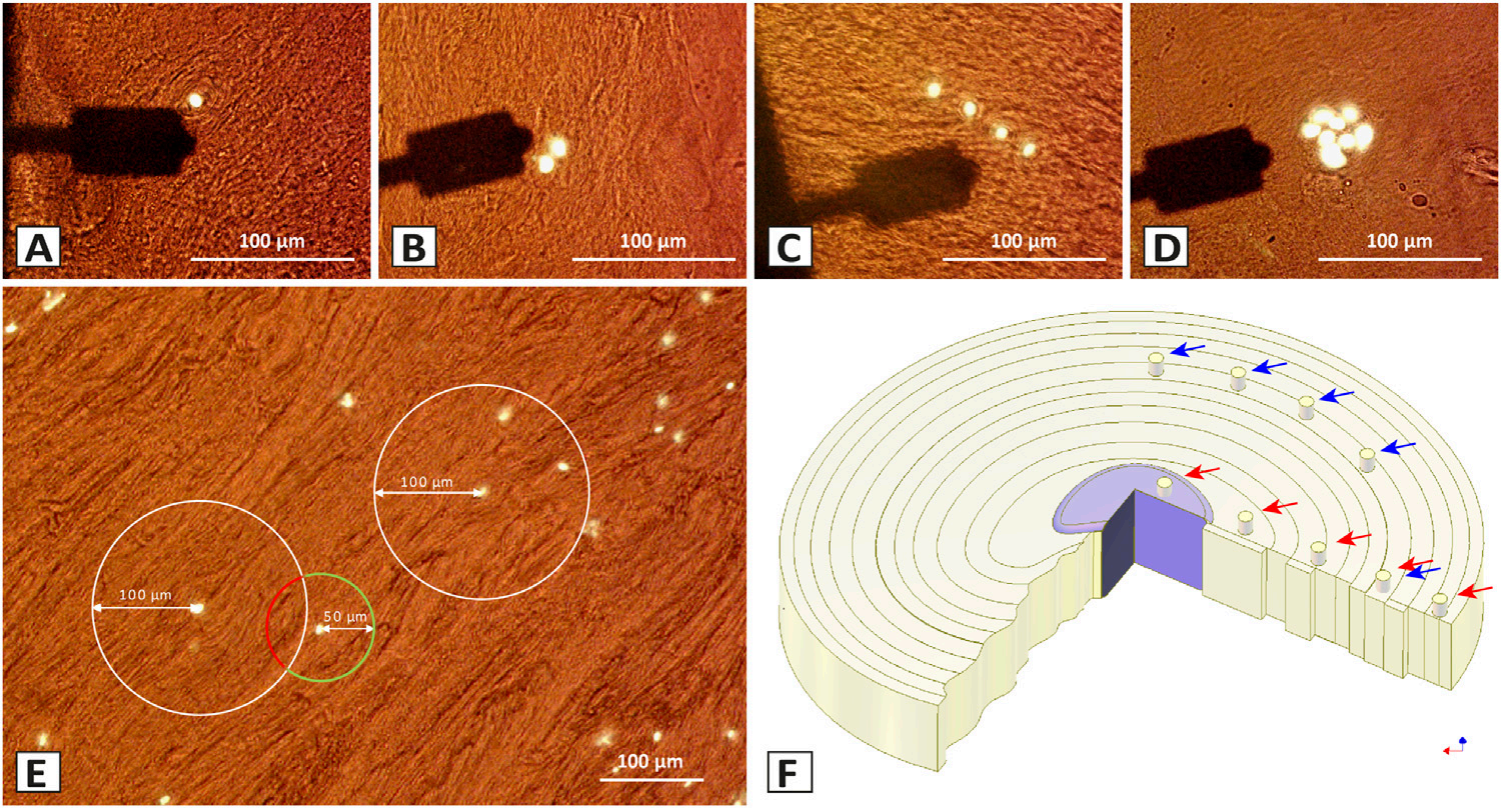
Different spatial chondrocyte patterns in the IVD, selection of the measurement sites, and sampled areas in the bovine IVD for the ECM and PCM using Atomic Force Microscopy (AFM). **(A–D)** Brightfield histological section of the IVD (35 µm) showing the arm of the cantilever (black) and the chondrocytes in their ECM (brown). The different spatial chondrocyte patterns analysed were single cells (SC) **(A)**, pairs (P) **(B)**, string-formations (SF) **(C)**, and clusters (Cl) **(D)**. The PCM measurement site was chosen directly over the nuclei (white). **(E)** The ECM measurement was performed at 50 µm away from the nuclei of the pattern of interest (green circle) and at the same time being at least 100 µm apart from all other patterns (determined by the white circle). **(F)** To evaluate the variability of the data with our measurement technique, tissue from five similar but independent locations in the central anulus fibrosus in one bovine IVD were analysed (blue arrows). To investigate the changes in ECM and PCM values and their ratio depending on their location in the disc, five different tissue origins (red arrows) were selected on each of five different bovine discs going from the central nucleus pulposus (CNP), to the inner intermediate zone (IIZ), the outer intermediate zone (OIZ), the inner anulus fibrosus (IAF), to the outer anulus fibrosus (OAF).

In articular cartilage, the degree of local tissue degeneration can be graded by its chondrocyte spatial organisation (Felka et al., 2016). Interestingly, the stiffness of both ECM and PCM decrease to a significant and comparable degree alongside the spatial pattern changes present with advancing degeneration (Danalache et al., 2019a; Danalache et al., 2019b; Tschaikowsky et al., 2020). In all different patterns, the PCM of the articular cartilage has been described to be softer than the ECM (by about 50%), thus possibly acting as a cushion around the cells during compressive loading. Drawing an analogy to articular cartilage, we found that chondrocytes in the healthy IVD are predominantly organised as single cells or pairs, but also string-formations. Similar to articular cartilage, in the process of degeneration cluster formation occurs (Roberts et al., 1991; Ciapetti et al., 2012; Bonnaire et al., 2021) (Figures 1A–D). Whether the presence of these different spatial patterns also has a direct functional implication in the IVD remains, to date, unknown. Atomic force microscopy (AFM) has already been successfully used to investigate the impact of degenerative changes at the nanoscale in the IVD (Lewis et al., 2008; Ashinsky et al., 2019; Cauble et al., 2020). To the best of our knowledge, no study has yet so far investigated the functional biomechanical implications of the cellular spatial organisation in the IVD.

Aim of the present study was to evaluate the stiffness of the ECM and PCM around anulus fibrosus chondrocytes as a function of cellular spatial organisation. To this end, we employed AFM measurements. Anulus fibrosus was chosen because of its high collagen content (65%) (Berthet-Colominas et al., 1982), that is comparable to collagen composition within the articular cartilage (60%) (Sophia Fox et al., 2009). Its structural integrity is essential for nucleus confinement while concomitantly maintaining physiological intradiscal pressures under loading (Chu et al., 2018). The anulus fibrosus has a unique lamellar structure, with seven to fifteen concentric layers (Humzah and Soames, 1988; Urban and Roberts, 2003; Baldit, 2018) visible on a macroscopic scale, allowing for easy and correct identification of the tissue structure. We hypothesised that there is no difference in matrix stiffness between single cells, pairs, and string-formations but that stiffness is lower around chondrocytes organised in clusters (indicative for degenerated tissue) (Hypothesis I). Based on findings from articular cartilage (Danalache et al., 2019b), we also hypothesised that the ECM/PCM ratio is constant across all different spatial patterns (Hypothesis II).

## Materials and methods

### Tissue sample acquisition

For this experimental and basic research study, 30 IVD samples were obtained from patients undergoing spine surgery including both dorsal lumbar fusion and sequestrectomy at the Department of Orthopaedic Surgery of the University Hospital of Tübingen, Germany. Full departmental, institutional, and local ethical committee approval were obtained (project number BO2925/2020) before commencement of the study. Informed consent was obtained from all subjects involved in the study. The parameters recorded were patient age at operation, sex, anatomic level of tissue origin, the Pfirrmann-score for MRI grading of IVD degeneration (Pfirrmann et al., 1976), and the type of procedure performed (Table 1).

**TABLE 1:** Characteristics of the investigated human IVD samples.

Variable	Number	
Analysed IVDs	30	
Patient age [year] (median/range)	56.5 (31–86)	
Sex	M	13
	F	17
Level	L2/3	1
	L3/4	4
	L4/5	16
	L5/S1	9
Pfirrmann score	2	1
	3	10
	4	17
	5	2
Procedure	Fusion	19
	Sequestrectomy	11

To describe the relationship of ECM and PCM in a healthy IVD environment, six bovine IVDs and one IVD obtained from a 14-year-old patient operated for scoliosis were additionally measured. Reasons for the choice of bovine disc tissue were the high loads present in this animal model and the loss of notochordal cells in the nucleus pulposus during maturation which is a feature also present in humans (Daly et al., 2016). The bovine samples were resected from young adult animals (18–24 months) purchased from a local abattoir.

### Tissue sample preparation

**FIGURE 2:**
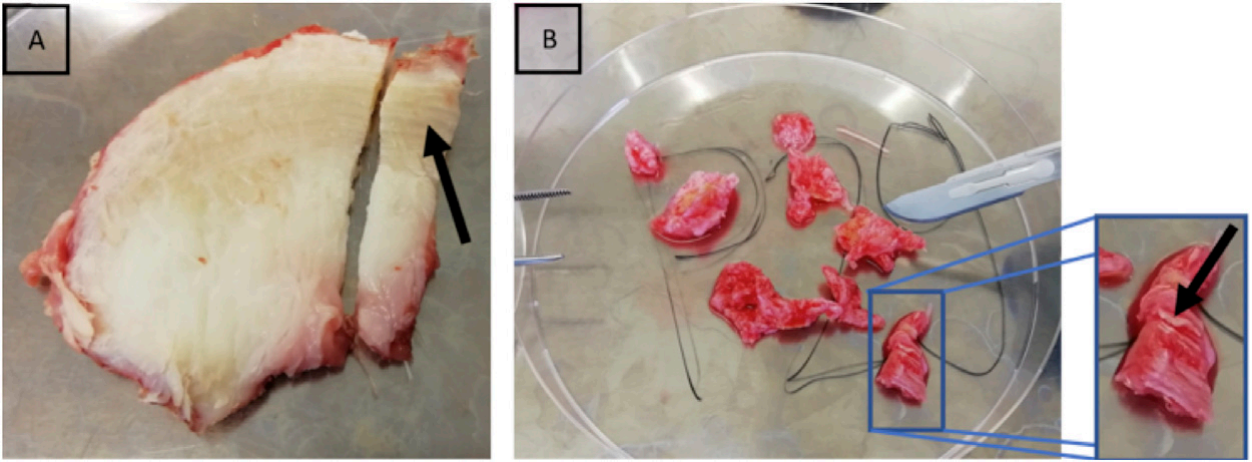
Selection of anulus fibrosus from intraoperatively obtained tissue. Lamellar structure of the anulus fibrosus. The black arrows point at the lamellar structure of the anulus fibrosus in bovine **(A)** and human IVD samples **(B)**. This lamellar structure can already easily be recognised by the naked eye.

Human tissue was temporarily stored after surgical resection in serum-free Dulbecco’s modified Eagle’s medium (Gibco, Life Technologies, Darmstadt, Germany) with 2% (v/v) penicillin-streptomycin and 1.2% (v/v) amphotericin B at room temperature. Further processing and measurements were carried out within 24 h of surgery, and the samples were fully immersed in DMEM to reduce the possibility of artifacts caused by dehydration and drying (Erisken et al., 2010). Due to the surgical procedure, the samples were received as fragments (<1 cm × 1 cm). From these tissue fragments, only areas with a clearly identifiable anulus fibrosus collagen architecture were selected (Figure 2). Only one sample per human patient was analysed. Samples were embedded in water-soluble embedding medium (Tissue-Tek O.C.T. Compound, Sakura Finetek, Alphen aan den Rijn, Netherlands) and sectioned with 35 µm thickness (Leica cryotome type CM3050S, Leica Biosystems, Wetzlar, Germany). The slices were rinsed with phosphate-buffered saline to remove the water-soluble embedding medium and they were then either prepared for later immunohistochemical analyses or AFM.

The bovine discs were harvested from the animals within 24 h after death and kept frozen until further processing. To evaluate the variability of the data acquired with the technique used, five independent tissue sample were resected from the same bovine disc at different locations in the bovine anulus fibrosus at a similar radial position. To evaluate how much results depend on the location of the tissue origin with the disc, five samples were measured along the radius of the disc from the inner nucleus to the outer anulus in each of five different bovine IVDs. The rest of the tissue preparation was carried out as in human tissue.

### Stiffness assessment of the extracellular matrix and pericellular matrix *via* atomic force microscopy

Prepared sections were fixed onto tissue culture dishes for AFM (TPP Techno Plastic Products AG, Trasadingen, Switzerland) with a biocompatible sample glue (JPK Instruments AG, Berlin, Germany). After rinsing them again with phosphate-buffered saline, they were dyed with a green nucleic acid stain (Sytox Green, ThermoFisher Scientific, Waltham, MA, United States) at a final concentration of 1 µM and covered with Leibovitz’s L-15 medium without L-glutamine (Merck KGaA, Darmstadt, Germany) for the measurements.

Elastic moduli (E) of the ECM and PCM were assessed as previously described by Danalache et al. (2019a), Danalache et al. (2019b). The used AFM system (CellHesion 200, Bruker, Berlin, Germany) is integrated into an inverted phase contrast microscope (AxioObserver D1, Carl Zeiss Microscopy, Jena, Germany). For microscale indentation, a cantilever with a spherical tip of 5 µm (Model: SAA-SPH-5UM, *k* = 0.2 N/m, Bruker, Billerica, MA, United States) was used. The calibration of the cantilever was done exactly as previously described by Danalache et al. (2020). In brief, the spring constant was calibrated using the thermal noise method in fluid (Ma et al., 2021). On the calibration force-distance-curve the region for linear fit of the extended curve was used for calibration as well as for Young’s modulus calibration.

The elastic properties of the ECM and PCM in relation to the different cellular patterns (Figures 1A–D) were assessed by performing indentations over a chosen pattern of interest identified by cellular fluorescence staining. While in healthy IVD tissue chondrocytes are predominantly organised as single cells or pairs but also string-formations can be found, in the process of degeneration cluster formation occurs (Bonnaire et al., 2021).

For the PCM, the nuclei were defined as the central targeting site for bead placement in the measurements. The ECM measurement site was defined as being 50 µm away from the nuclei of the pattern of interest and at the same time being at least 100 µm away from all other patterns (Figure 1E). Only chondrocytes lying proximally to the section surface than the centre of the section while at the same time still being fully covered with matrix were measured so as to measure the cell-PCM-ECM interface with a maximum ECM coverage of less than 6 µm (35 µm sections, cell diameter around 12 µm). To determine stiffness, E—also known as Young’s modulus—was calculated from the force-distance curves by using the Hertz model incorporated into the data processing software (Bruker, Berlin, Germany). The calculations were performed as described in Eqs 1, 2, with F, Force, E, Young’s Modulus, v, Poisson’s ratio, *δ*, indentation, *a* = radius of contact circle, and Rs, radius of the used microsphere.

$$F=\frac{E}{1-v^{2}}\;\left[\frac{a^{2}+R_{s}^{2}}{2}\ln\frac{R_{s}+a}{R_{s}-a}-aR_{s}\right]$$ (1)

$$\delta =\frac{a}{2}\ln\frac{R_{s}+a}{R_{s}-a}$$ (2)

The Poisson ratio was set as 0.5 (Jin and Lewis, 2004; Choi and Zheng, 2005; Thambyah et al., 2006) and the indentation depth was about 1 µm i.e., 3% of the sample thickness.

### Immunohistochemical analyses

Sections were mounted without fixation and encircled with a lipid layer using Super Pap Pen (Science Services) to allow staining on the slide. Histological sections were pretreated with 0.2% (w/v) collagenase type XI (Sigma‐Aldrich, Taufkirchen, Germany) for collagen type VI staining and with 0.1% (w/v) hyaluronidase (Sigma‐Aldrich) for collagen type III, perlecan, and biglycan staining in PBS for 3 h at 37°C, followed by three washing steps with PBS. To reduce unspecific antibody binding, sections were blocked for 60 min in 5% (w/v) bovine serum albumin and 0.3% (v/v) Triton X‐100 in PBS. This was followed by incubation with the primary monoclonal antibodies at a dilution of 1:100 in 2.5% (w/v) bovine serum albumin‐PBS at 4°C overnight: collagen type VI (rabbit anticollagen VI, ab‐182744; Abcam, Cambridge, United Kingdom), collagen type III (rabbit anti‐collagen type III, ab‐7778; Abcam), perlecan (mouse anti‐perlecan, sc‐377219; Santa Cruz Biotechnology Inc., Dallas, TX), and biglycan (rabbit anti‐byglican, HPA003147; Sigma‐Aldrich). Sections were rinsed with PBS and incubated with secondary antibodies at a dilution of 1:100 (Alexa Fluor 555 goat anti‐rabbit immunoglobulin G [IgG], a‐21429; Thermo Fisher Scientific, or Alexa Fluor 594 goat anti‐mouse IgG, ab‐150116; Abcam) for 2 h in complete darkness. Nuclei of the cells were stained with 4′,6‐diamidino‐2‐phenylindole (DAPI) (Life Technologies, Darmstadt, Germany) at a dilution of 1:1000 in PBS for 5 min. Fluorescent staining was visualized with a Carl Zeiss Observer Z1 fluorescence microscope (Carl Zeiss Microscopy, Jena, Germany) equipped with MosaiX image acquisition software (Carl Zeiss Microscopy).

### Statistical analysis

For every IVD sample, three independent patterns for each of the four different pattern types (single cells, pairs, string-formations, clusters) (Figures 1A–D) were measured. On each pattern two sites were measured for the ECM and two for the PCM. Nine measurement repetitions were performed on each measurement site, as previously described (Danalache et al., 2019b). In total, per disc sample, 432 measurements were performed. In the main study group, after computing the median of the nine measurement repetitions of every measurement site, the arithmetic mean of the two medians of every individual pattern was calculated. Hereupon the arithmetic mean of the three independent patterns of the same type was generated to obtain one value per pattern type and matrix type. In the end, eight values per patient were included in the final statistical evaluation, specifically: single cells-ECM, single cells-PCM, pairs-ECM, pairs-PCM, strings-ECM, strings-PCM, clusters-ECM, clusters-PCM. Normality was assessed my means of histograms and Shapiro-Wilk test and a non-parametric approach was chosen. Data are reported as median and interquartile range (IQR) and are displayed as boxplots. The interconnection of ECM and PCM was evaluated by their ratio based on individual ratios calculated from the final means of each measurement site for the ECM and the PCM. Comparison of the different groups was performed by Kruskal-Wallis test using the Mann-Whitney *U* test for post-hoc testing. All *p*-values were calculated on a two-tailed basis with the exception for stiffness comparison of clusters with the other patterns. In this case we chose a one-tailed analysis since we clearly expected values of clusters to be lower than in the other groups. This assumption is supported by the fact that cell clusters are a hallmark of degeneration found in a wide variety of cartilaginous structure such as articular cartilage (Lotz et al., 2010), intervertebral disc (Tolonen et al., 2002; Sowa et al., 2008; Zhao et al., 2008), meniscus (Hellio Le Graverand et al., 2001), and cricoarytenoid cartilage (Paulsen and Tillmann, 1999), which is also associated with a significant loss of stiffness (Danalache et al., 2019b; Tschaikowsky et al., 2021). Sample size calculation was performed taking a one-sided inferential approach matching with our hypothesis and based on a Type I error rate (alpha) of 0.05 and a Power (1-beta) of 0.8. The ratio of first samples to second samples equals 1 and this factor can be completely controlled for. The mean of the measurements for the PCM with our measurement technique was 1.89 kPa in the bovine anulus tissue which we used as exploratory experiment. The evaluated standard deviation for these values was 0.79 kPa. In our own studies in cartilage, the actual observed difference between the healthy single strings and big clusters exceeded by far 100%. Setting, however, the minimal difference between healthy patterns and clusters in the anulus fibrosus which we would consider still relevant at 30% impairment of the stiffness, this would result in a mean stiffness of clusters of 1.32 kPa. To demonstrate such a difference as being statistically significant, this would require a sample size of *n* = 23 in each of the groups. To account for unexpected variables and slightly larger group with *n* = 30 was investigated.

For the bovine tissue and the 14-year-old human IVD, due to the low sample size, all medians of the nine measurement repetitions per measurement site were used for further analysis thus treating all medians as an independent value. This assumption could be made as stiffness values vary greatly spatially (Ho et al., 1976). Only single cells and double cells were measured in these samples and the results are presented taking both patterns together. Statistical analysis was performed with SPSS Statistics 22 (IBM Corp., Armonk, NY, United States).

## Results

**FIGURE 3:**
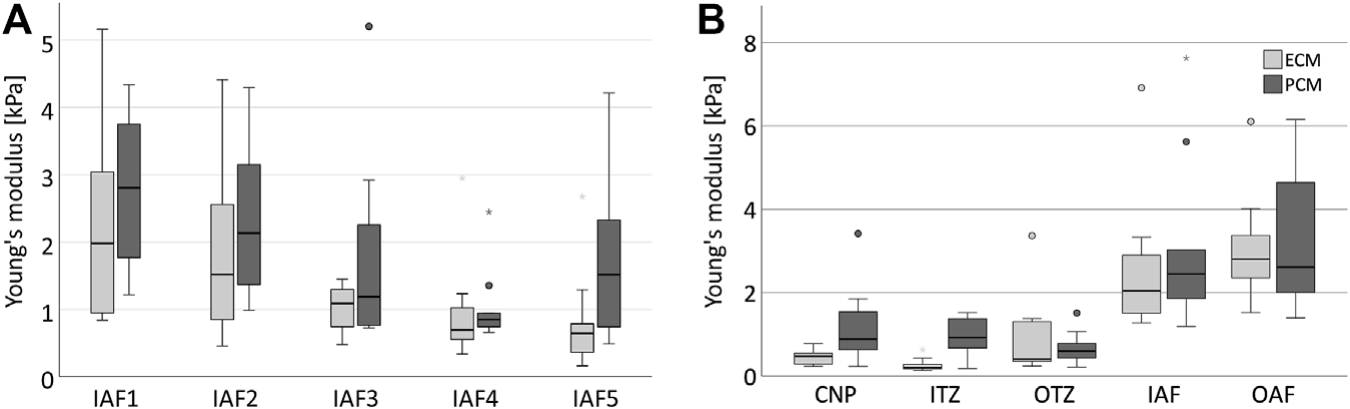
Stiffness depends on the localisation in the disc. Boxplots showing the results for ECM (light grey) and PCM (dark grey) measurements for five similar measurement areas in one single anulus fibrosus **(A)** and for five different disc zones going from the centre to the periphery of the IVD measured in five different bovine discs **(B)**. **(A)** It can be noted that a relevant variability of the data is measured even in the same type of tissue in the same disc (ECM: *p* = 0.001, PCM: *p* < 0.001, ECM/PCM ratio: *p* = 0.002). The scope of variability is, however, comparable for each ECM and PCM testing within one sample. **(B)** The stiffness of ECM and PCM appears to increase from the central disc to the outer anulus. Abbreviations: CNP, central nucleus pulposus; IIZ, inner intermediate zone; OIZ, outer intermediate zone; IAF, inner anulus fibrosus; OAF, outer anulus fibrosus.

Prior to the actual measurements, one bovine disc was assessed to evaluate the suitability of the technique and the data variability to estimate a suitable sample size. When measuring stiffness of the ECM and PCM at five different locations of seemingly similar tissue (Figure 1F) we observed a relevant variation (roughly by a factor of 3) in the data (*p* = 0.001 for the ECM, *p* < 0.001 for the PCM) (Figure 3A) which led to the high sample size we chose to evaluate the human disc. To explore possible differences between nucleus and anulus, we performed measurements on five different locations in each of five bovine discs going from the centre to the outside (Figure 1F).

**FIGURE 4:**
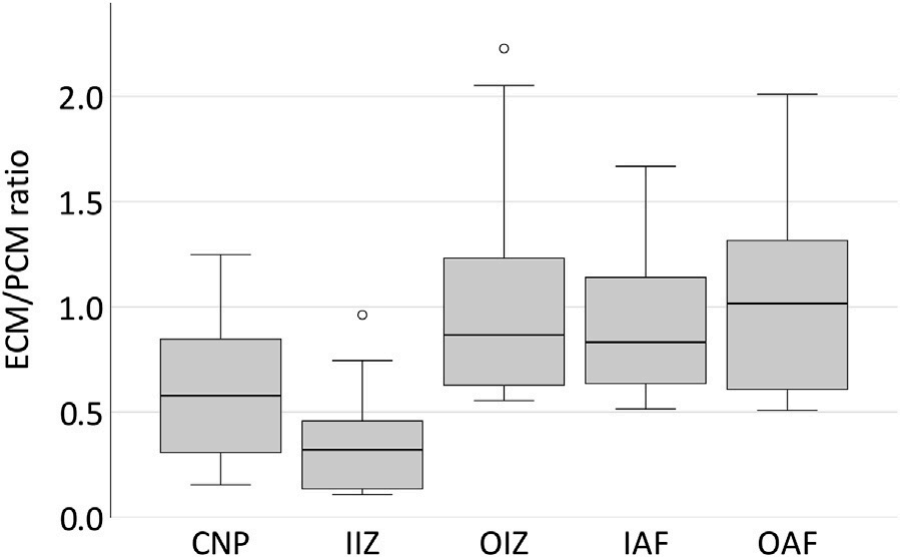
The PCM in the bovine IVD is stiffer than the ECM in central areas of the disc while comparable in size in the outer parts. Boxplots showing the ECM/PCM ratio of the Young’s modulus for the different locations in five different bovine discs from the centre to the periphery. It can be noted that while in the outer areas of the disc ECM and PCM are of a comparable order of magnitude, the PCM is much stiffer than the ECM in the centre of the disc with a ratio of 0.58 for the central nucleus pulposus and even 0.32 for the inner intermediate zone. Abbreviations: CNP - central nucleus pulposus; IIZ - inner intermediate zone; OIZ - outer intermediate zone; IAF - inner anulus fibrosus; OAF - outer anulus fibrosus.

Both ECM and PCM stiffness values were higher in the outer areas of the disc than in the centre (*p* = 0.002, Figure 3B). When looking at the relationship of ECM and PCM values, we surprisingly noted that values for the ECM were actually significantly lower than for the PCM in the centre of the disc (*p* = 0.029 for the nucleus pulposus and *p* = 0.002 for the inner intermediate zone) while being comparable in size in the outer areas (Figure 4).

**FIGURE 5:**
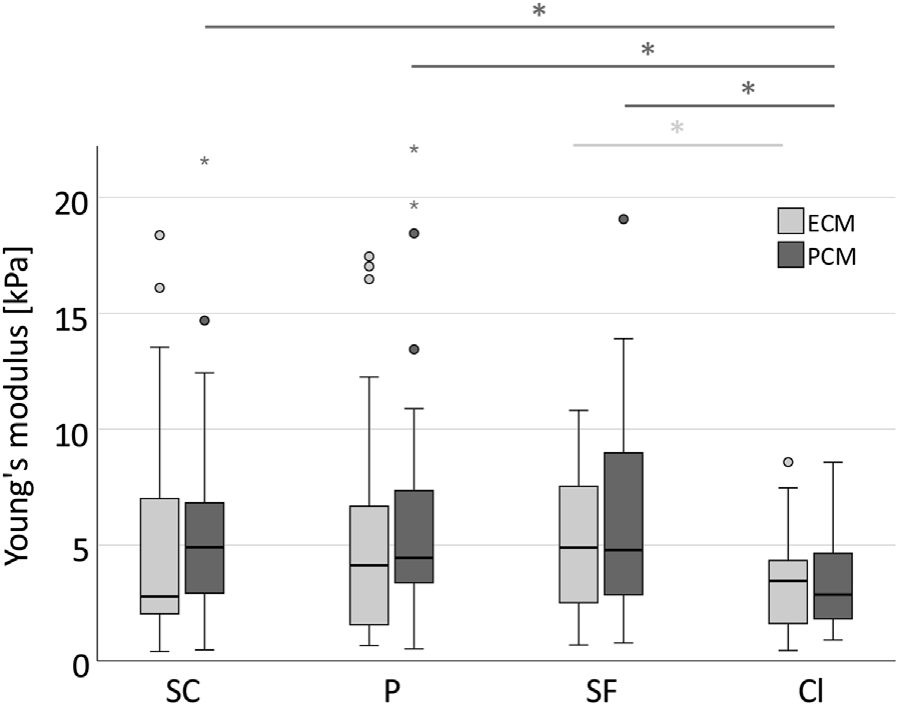
Stiffness in clusters is significantly reduced in the PCM compared to physiological spatial chondrocyte patterns. Boxplot displaying the Young’s moduli for the ECM (light grey) and PCM (dark grey) across the different spatial chondrocyte patterns in the human degenerate IVD. While measurement results showed no difference between single cells, pairs, and string-formations, stiffness was significantly lower in chondrocytes arranged in clusters for the PCM (SC -Cl: *p* = 0.026, P-CL: *p* = 0.016, SF-Cl: *p* = 0.010). The values for the ECM in clusters failed to reach statistical significance with the exception of the difference between string-formations with clusters (*p* = 0.022). No relevant difference was observed between values for the ECM and the PCM. Abbreviations: SC, single cells; P, pairs; SF, string-formations; Cl, clusters. *p < 0.05.

**FIGURE 6:**
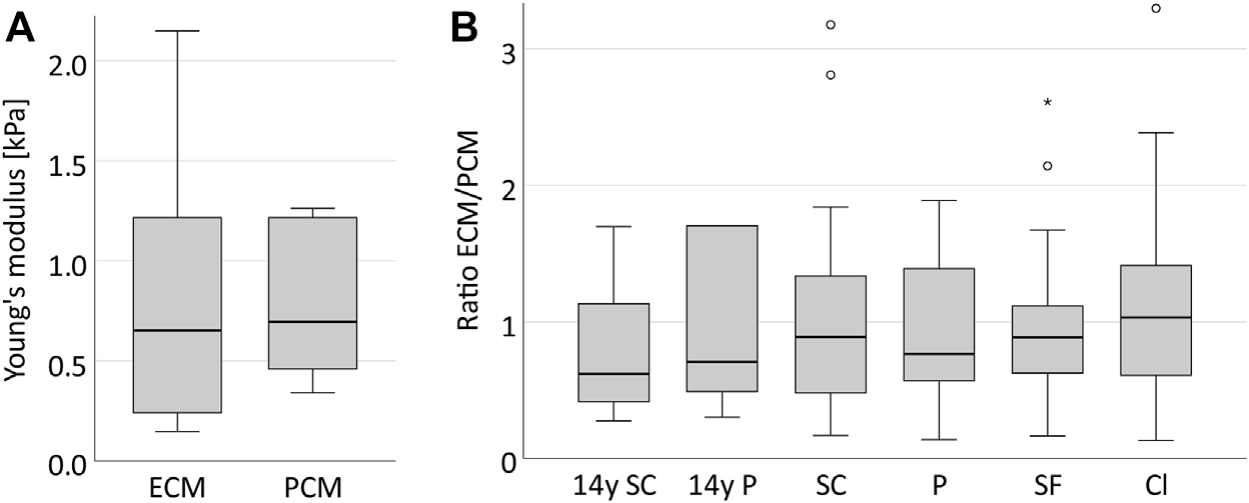
The ECM and PCM in the human IVD present with a similar stiffness and their ratio remains constant across all spatial chondrocyte patterns. Boxplots illustrating the stiffness of the ECM and PCM in one supposedly healthy anulus fibrosus sample from a 14-year-old patient **(A)** and its ECM/PCM ratios including also all samples and patterns of the collective with degenerative IVDs **(B)**. It can be observed that the stiffness of both matrices is very similar (*p* = 0.458) **(A)** and that their ratio remains constant around the value of 1 in the course of IVD degeneration (*p* = 0.758) **(B)**. Abbreviations: SC, single cells; P, pairs; SF, string-formations; C, clusters; y, years.

In the human anulus fibrosus, as expected, we found no difference between the three supposedly healthy spatial chondrocyte patterns (single cells, pairs and string-formations, Figure 5). PCM stiffness was, however, significantly lower in clusters than in the other three patterns (Figure 5). The ratio of ECM and PCM stiffness showed no relevant variability across all spatial patterns (*p* = 0.758) including the tissue sample from a 14-year-old patient, with all values being slightly lower than 1 apart from clusters where the ratio was 1.03 (Figure 6).

**FIGURE 7:**
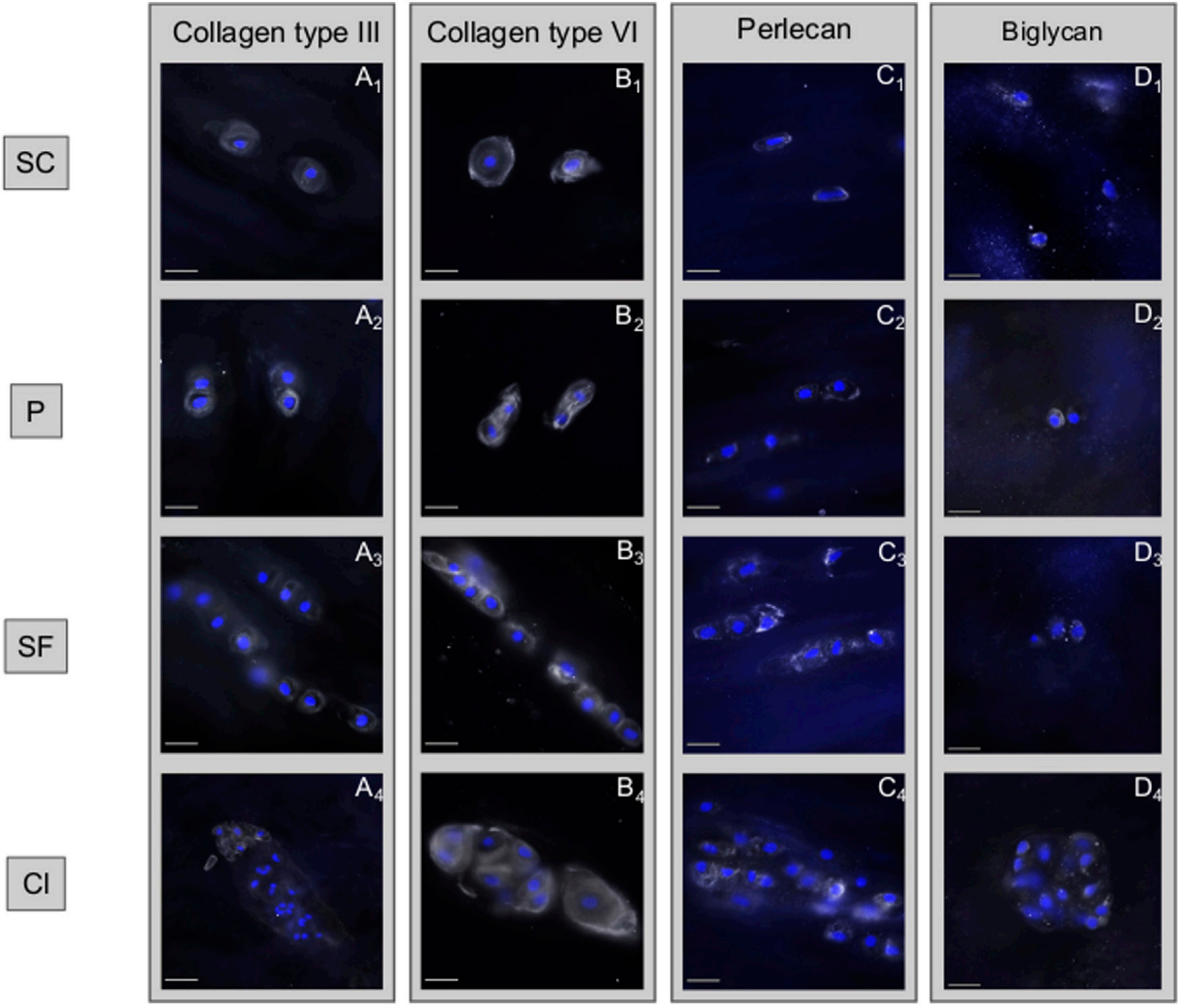
Disruption of collagen type III and softening of the pericellular matrix are observed in chondrocyte clusters. Immunohistochemical analyses of different matrix components of the intervertebral disc based on the different spatial cellular patterns. **(A)** Collagen type III is firmly present in the healthy pericellular microenvironment of single cells (SC), pairs (P) and string-formations (SF) while it is mostly absent in clusters (Cl). **(B)** While collagen type VI is constantly present in all spatial patterns, being a marker for the pericellular matrix, it highlights the loss of structural confinement of this structure. **(C)** This loss of spatial confinement appears also for perlecan. **(D)** The ubiquituously present matrix proteoglycan biglycan remains largely unaffected by the changes taking place. **(A–D)** describes different subtypes of matrix components that are present in the matrix, the numbers 1–4 describe how each of these matrix components changes with time. Abbreviations: SC, single cells; P, pairs; SF, string-formations; C, clusters.

Immunohistochemical analyses showed that collagen type III is firmly present in the healthy pericellular microenvironment of SC, P, and SF while it is mostly absent in Cl. The PCM also appears less well defined and confined in Cl as can be observed in the collagen type VI staining (Figure 7).

## Discussion

Using AFM, we evaluated the stiffness of the ECM and PCM in the anulus fibrosus of the IVD based on spatial organisation of the chondrocytes. As expected, we found no difference in stiffness between single cells, pairs, and string-formations but significantly lower values for the PCM around chondrocytes organised in clusters (by approximately 40%). In articular cartilage, the E of both the ECM and the PCM decreases continuously from strings to double strings, to small and then to big clusters in the course of degeneration (Danalache et al., 2019a; Danalache et al., 2019b; Tschaikowsky et al., 2020). These four spatial patterns present a physiopathological sequence from still largely intact to highly disrupted tissue. To date, such a sequence has not been described in the IVD. Since three different physiological organisational patterns seem to be present in the IVD (single cells, pairs and string-formations) (Bonnaire et al., 2021), a clear intermediate step to clusters is hard to define. Moreover, in a normal joint there are always areas with higher loads and areas that can avoid most of the stress. It is in these areas, that even in advanced osteoarthritis still relatively healthy cartilage can be found. In contrast, in the IVD, all areas of the joint are under high loads leading to a partial disappearance of degenerated tissue in the compromised disc, thus possibly explaining the lack of presence of monstrous clusters that are regularly found in osteoarthritic articular cartilage (Lotz et al., 2010). Nevertheless, the fact that in areas with clusters PCM stiffness is significantly reduced when compared to areas with one of the three healthy spatial patterns is remarkable: although measurements for all patterns were taken on the same discs and even the same histologic section thus being in close proximity, the fact that chondrocytes had changed their spatial pattern appears to be enough to be associated with relevantly different biomechanical properties. Similarly, in clusters the ECM was also characterised by low stiffness. This reduction failed, however, to reach statistical difference and thus needs to be further investigated.

The fact that these local differences in matrix stiffness are present suggests a difference in catabolic state of the chondrocytes. Cell clusters, which are most common in degenerate IVDs, are marked by an altered protein and gene profile (Sive et al., 2002; Sharp et al., 2009; Gan et al., 2021). This may either lead to a locally more disrupted matrix synthesis to repair the damaged matrix or to increased local tissue degradation orchestrated by upregulated enzymatic activity (Furtwangler et al., 1976; Le Maitre et al., 2004). It is already known that during disc matrix turnover, development, and degeneration, the IVD chondrocytes produce a wide sequel of matrix metalloproteinases, responsible for degrading matrix components (Chen and Jiang, 2021). In this context it appears highly interesting, that collagen type III largely disappears from the cellular microenvironment in clusters. This loss in structure protein may explain the measured reduction in local tissue stiffness. In addition, the PCM appears also less confined in clusters, which would be in line with the impression that the elasticity ratio of ECM/PCM does not decrease with clusters. Despite its localisation mostly in the PCM, a key role of collagen type III for both ECM and PCM stiffness has been proposed recently derived from AFM measurements from articular cartilage and meniscus {Wang, 2020 #67}. It was suggested that collagen type III plays a critical role in mediating fibril assembly (Wang et al., 2020), which would thus directly impact biomechanical functions of both the PCM and ECM.

Although in articular cartilage the PCM is much softer than the ECM thus possibly acting like a cushion under high physiological loading (Danalache et al., 2019a; Danalache et al., 2019b), no difference was found in the anulus fibrosus when comparing the PCM (close proximity to the cells) to the ECM (at 50 µm distance). This observation may be explained by three possibilities: First, it is conceivable that we did in fact not indent the PCM exclusively. Assuming a cell diameter of about 10–15 µm (Guilak et al., 1999) and a cell position in the top half of the section this would mean a coverage with PCM/ECM of only a few µm. At a described thickness of the PCM of 2.5–4 µm in the anulus (Cao et al., 2007) we might have measured the stiffness of the chondrocytes instead. In such a scenario the chondrocytes would present with an identical stiffness as the ECM which would already be an interesting finding by itself. The observation that when using the same setup in the bovine disc, the PCM showed significantly higher stiffness values than the ECM only in the central portion of the disc, argues against the possibility that the coverage of the chondrocytes by some ECM simply leveled out all possible differences that might be present underneath. Also, the finding that the ECM/PCM stiffness ratio is constant across all patterns while absolute values for both matrixes are largely reduced in clusters than in the other three patterns argues against such a possibility. If it were indeed the cells that we measured, this would mean that chondrocyte stiffness decreases exactly in the same degree in clusters as does the ECM, which we consider unlikely. The second possibility is that the structure known as the PCM in articular cartilage is not really present in the anulus fibrosus. There are numerous studies describing the PCM in articular cartilage (Wilusz et al., 2014; Chu et al., 2017; Danalache et al., 2019a) and several studies illustrating the existence of the PCM in the nucleus pulposus (Prabhu et al., 2005; Roberts et al., 2006; Cao et al., 2011). There is also literature available describing the existence of the PCM in the anulus fibrosus (Gruber et al., 2007; Hofmann et al., 2018). In these works, the PCM appears, however, morphologically different in the dense collagen type I lamellae architecture than in the collagen type II mesh of articular cartilage or the nucleus. For this reason, as a third explanation, the function of the PCM in the IVD may be of a different nature than in articular cartilage—and probably even differs within the disc between nucleus and anulus. In fact, while the PCM of the anulus fibrosus appears to be very thin and encompasses mostly individual chondrocytes (Cao et al., 2007), in the nucleus pulposus more than half of nucleus pulposus chondrocyte-like cells have been found to reside in a common PCM shared by multiple cells concomitantly (Cao et al., 2007; Cao et al., 2011). At least in the intact IVD, the compressive forces present in the disc mostly act on the nucleus pulposus. In this zone of the bovine samples, our exploratory results hint that the PCM is even much stiffer than the ECM. In the anulus fibrosus, both matrixes showed an approximately equal degree of stiffness. Here, the compressive forces from the nucleus are translated to tensile forces thus creating massive shear stress. If looking for a comparable situation in articular cartilage, this might be in the topmost layer, where actually the PCM also looks much different than in the deeper zones (Wilusz et al., 2014). Different functional properties have also been suggested for the PCM in the superficial zone of articular cartilage than in the deeper layers (Choi et al., 2007; Julkunen et al., 2009). In unconfined IVD loading studies that really created shear stress on the disc tissue, it could be shown that the PCM is protective for the cells (Hofmann et al., 2018). It can thus be speculated here, that it is indeed a different function of the PCM in the anulus fibrosus compared to the nucleus or the articular cartilage that yields these results. We will carry out further confirmatory studies on this hypothesis.

Degeneration of the tissue affects the IVD on all levels which thus also leads to disruption of the IVD matrix (Urban and Roberts, 2003). It remains unknown, however, whether this matrix degradation preferentially affects the ECM or the PCM (Hypothesis II). Despite the changes in values of the Young’s modulus for both structures from the healthy cellular patterns (single cells, pairs, string-formations) to clusters indicative of degenerated tissue (Johnson et al., 2001) no statistically significant changes in the ECM/PCM ratio could be observed. Looking at the relationship of stiffness of the ECM and the PCM, it could indeed be observed that the ratio of these two structures is very similar throughout all different spatial patterns. This implies that the changes of the ECM and the PCM occur unidirectionally, simultaneously and to a comparable degree, suggesting that the underlying destructive mechanisms are of the same nature. Our results presented here are thus in line with those from articular cartilage (Darling et al., 2010; Danalache et al., 2019b).

## Study limitations

Especially the PCM is a structure that is difficult to biomechanically measure in absolute terms since its elasticity is driven by its interconnection with the surrounding ECM and the encompassed cells with their cytoskeleton. Only functional measurements can describe its biological function. Absolute values presented in this study have to be interpreted with care as they may vary depending on various experimental parameters used for mechanical testing, such as indentation velocity and depth, indenter shape and size, and accurate representation of tip geometry in model fitting (Costa and Yin, 1999; Stolz et al., 2004; Park et al., 2009; Qian and Zhao, 2018). This should not, however, affect the results within one study and their relation to each other. Even though throughout the entire sample preparation procedure special attention was payed to avoid sample dehydration by placing the samples always in appropriate medium as previously suggested by Erisken et al. (2010), we cannot fully exclude possible artefacts due to loss of water or viscous content.

Since a relevant variability in the data cannot be avoided (Figure 3), a highly standardised approach with a sufficiently large sample size was employed in humans to obtain statistically reliable results. The results from our collective only reflect the situation in the anulus as no human nucleus tissue was measured. AFM results always strongly depend on the setup chosen for the experiments. While a 5 µm bead, as used in this study, might be ideal to measure the PCM due to its cocoon-like circumferential anatomy, it is conceivable that the ECM results depend on whether a collagen lamellae is directly hit or whether measurements are taken in a more proteoglycan rich area. We do not see, however, differences in variance of ECM and PCM measurements arguing against such an assumption. PCM-measurements directly on the chondrocytes might be also influenced by the stiffness of the cells themselves when compared to measurements in proximity to the cells in the *x*-*y*-axis. On the other hand, in the latter technique such a confounding factor would be the ECM. Moreover, our way of measuring the PCM maintains PCM integrity, which we believe to be of critical importance since its cocoon-like structure is functionally completely disrupted when injured during sectioning. As a result of this technique, our measured values can only be interpreted in relation to other values obtained by this study and do not possess absolute characteristics. All samples were received from patients undergoing a surgical intervention for scoliosis, lumbar disc degeneration or slipped disc. Thus, the “healthy” reference tissue (14-year-old patient) employed in the study was also derived from an IVD which might have been altered by the condition “scoliosis”. Nevertheless the cellular patterns we identified in our “healthy” sample are in line with the observations made by Bonnaire et al. (2021), that single cells are the predominant spatial patterns (>50%) in healthy bovine IVD samples.

When receiving tissue from lumbar fusion surgery, the anatomic origin of the tissue within the IVD cannot be determined. Only indirectly, by evaluating the structure of the tissue (i.e., presence of lamellae), such a determination is possible. Tissue that did not present this lamellar structure was not examined. Since the obtained results seem to depend on the location of the tissue in the disc, this factor needs to be balanced out by the sample size. Since ECM and PCM measurements were done on different patterns from the same histologic section, this problem should not affect the comparison of values between those patterns.

## Conclusion

This study is the first to describe and quantify the differences in stiffness the ECM in relation to the PCM on the basis of spatial chondrocyte organisation in the IVD. Despite being measured in end-stage disc degeneration in tissue areas very close to each other, a significant difference in the PCM stiffness between supposedly healthy spatial chondrocyte patterns (single cells, pairs and string-formations) and clusters was noted. Cluster formation is, thus, not only a morphological phenomenon describing disc degeneration but it marks a compromised biomechanical functioning. The ECM/PCM ratio remained almost unchanged alongside the changes in cellular spatial organisation, suggesting that the elastic changes of the two matrixes occur at a comparable degree.

## Data Availability

The raw data supporting the conclusion of this article will be made available by the authors, without undue reservation.

## Abbreviations

AFM, atomic force microscopy; ECM, extracellular matrix; E, elastic moduli; IVD, intervertebral disc; MRI, magnetic resonance imaging; PCM, pericellular matrix.
